# Utilization of adipocyte-derived lipids and enhanced intracellular trafficking of fatty acids contribute to breast cancer progression

**DOI:** 10.1186/s12964-018-0221-6

**Published:** 2018-06-18

**Authors:** Dejuan Yang, Yunhai Li, Lei Xing, Yiqing Tan, Jiazheng Sun, Beilei Zeng, Tingxiu Xiang, Jinxiang Tan, Guosheng Ren, Yuanyuan Wang

**Affiliations:** 1 Chongqing Key Laboratory of Molecular Oncology and Epigenetics, The First Affiliated Hospital of Chongqing Medical University, Chongqing, 400016 China; 2 Department of Endocrine and Breast Surgery, The First Affiliated Hospital of Chongqing Medical University, Chongqing, 400016 China

**Keywords:** Adipocyte, Breast cancer, Crosstalk, ATGL, FABP5, Aggressiveness

## Abstract

**Background:**

To determine whether adipocyte-derived lipids could be transferred into breast cancer cells and investigate the underlying mechanisms of subsequent lipolysis and fatty acid trafficking in breast cancer cells.

**Methods:**

A Transwell co-culture system was used in which human breast cancer cells were cultured in the absence or presence of differentiated murine 3 T3-L1 adipocytes. Migration/invasion and proliferation abilities were compared between breast cancer cells that were cultivated alone and those co-cultivated with mature adipocytes. The ability of lipolysis in breast cancer cells were measured, as well as the expression of the rate-limiting lipase ATGL and fatty acid transporter FABP5. ATGL and FABP5 were then ablated to investigate their impact on the aggressiveness of breast cancer cells that were surrounded by adipocytes. Further, immunohistochemistry was performed to detect differential expression of ATGL and FABP5 in breast cancer tissue sections.

**Results:**

The migration and invasion abilities of cancer cells were significantly enhanced after co-culture with adipocytes, accompanied by elevated lipolysis and expression of ATGL and FABP5. Abrogation of ATGL and FABP5 sharply attenuated the malignancy of co-cultivated breast cancer cells. However, this phenomenon was not observed if a lipid emulsion was added to the culture medium to substitute for adipocytes. Furthermore, epithelial-mesenchymal transaction was induced in co-cultivated breast cancer cells. That may partially due to the stimulation of PPARβ/δ and MAPK, which was resulted from upregulation of FABP5. As evidenced by immunohistochemistry, ATGL and FABP5 also had higher expression levels at the invasive front of the breast tumor, in where the adipocytes abound, compared to the central area in tissue specimens.

**Conclusions:**

Lipid originating from tumor-surrounding adipocytes could be transferred into breast cancer cells. Adipocyte-cancer cell crosstalk rather than lipids alone induced upregulation of lipases and fatty acid transport protein in cancer cells to utilize stored lipids for tumor progression. The increased expression of the key lipase ATGL and intracellular fatty acid trafficking protein FABP5 played crucial roles in this process via fueling or signaling.

**Electronic supplementary material:**

The online version of this article (10.1186/s12964-018-0221-6) contains supplementary material, which is available to authorized users.

## Background

Over the last few decades, substantial attention has been paid to the role of the microenvironment in tumorigenesis as well as tumor growth, invasion, survival and metastasis. The microenvironment of the breast is composed of the extracellular matrix and numerous stromal cell types, including endothelial and immune cells, fibroblasts and adipocytes [1]. Adipocytes, which were previously defined as an inert cell population that simply store lipids, are the predominant cellular component of the breast [2]. For a long time, the impact of adipocytes on cancer, particularly in fat-rich tissues, such as the breast, was not appreciated. In the last decade, epidemiological studies and pooled analysis have revealed positive associations between an increased body mass index, cancer morbidity and mortality [3]. The results caused alarm, and the link between obesity/adipocytes and cancer is now receiving serious consideration.

The review by Scherer et al. provides a summary of obese adipocyte-derived secretions and their tumor-promoting effects through paracrine pathways at the local level as well as via systemic endocrine pathways [4]. Numerous studies have been dedicated to unraveling the potential connections between various types of cancer cells and their surrounding adipocytes via messenger exchange, which ultimately supports the progression of cancer [5–9]. Reprogramming of energy metabolism is an emerging hallmark of cancer, and latent metabolic vulnerabilities have been identified from a therapeutic perspective [10, 11]. Adipose tissue is a large energy reservoir. Recently, a number of studies have elucidated the altered metabolism of fatty acids (FAs) in cancer cells alongside the impact of the modified profile of adipokines released by obese adipose tissue [4, 12]. The cancer cell-adipocyte interaction, which induces lipolysis in adipocytes and enables transfer of FAs from adipocytes into cancer cells, ultimately leading to β–oxidation in cancer cells, has been demonstrated in acute myeloid leukemia [13], ovarian and prostate carcinoma [5, 14]. The metabolic crosstalk between adipocytes and breast cancer cells is analogous to the reciprocal interaction between adipocytes and ovarian cancer cells or acute myeloid leukemia blasts. Balaban et al. and Wang et al. demonstrated lipolysis in adipocytes and a concomitant increase in FA oxidation (FAO) in breast cancer cells after co-culture of these two cell populations [15, 16]. However, some of the mechanisms involved in this metabolic crosstalk remain obscure. It is unclear how FAs precisely shuttle in intercellular and intracellular spaces as well as whether exogenous fat rather than adipocyte-derived lipids can serve as metabolic substrates for breast cancer cells. We anticipate that our results will complement other findings regarding the interaction between adipocytes and breast cancer cells.

## Methods

### Cell lines and plasmid/siRNA transfection

Murine 3 T3-L1 preadipocytes (generously provided by the Laboratory of Glucose and Lipid Metabolism, The First Affiliated Hospital of Chongqing Medical University, Chongqing) were cultivated and induced to differentiation as described by Liu et al. [17]. Immortalized normal breast epithelium MCF-10A cells (provided by the Key Laboratory of Animal Models and Human Disease Mechanisms of the Chinese Academy of Sciences and Yunnan Province, Kunming Institute of Zoology, Kunming) were cultured in Dulbecco’s modified Eagle’s medium (DMEM)/F12 medium (Gibco, Carlsbad, CA) supplemented with 5% horse serum (Gibco), 10 μg/ml insulin (Sigma–Aldrich, St. Louis, MO), 20 ng/ml EGF (Invitrogen, Carlsbad, CA), 100 ng/ml cholera toxin (Sigma–Aldrich) and 0.5 μg/ml hydrocortisone (Sigma–Aldrich). Breast cancer SUM159PT cells (obtained from the Tianjin Key Laboratory of Tumor Microenvironment and Neurovascular Regulation, Medical College of Nankai University, Tianjin) were cultivated as described by Forozan et al. [18]. The other breast cancer cell lines employed in this study were grown in RPMI 1640 medium containing 10% fetal bovine serum (both from Gibco). All cell lines were maintained at 37 °C in a 5% CO_2_ humidified atmosphere in medium supplemented with 1% antibiotics. Human adipose triglyceride lipase (ATGL)-targeting short hairpin RAN (shRNA) oligonucleotide sequences were cloned into psi-LVRH1P vector (synthesized by GeneCopoeia, FulenGen Co., Ltd., Guangzhou). A fatty acid binding protein 5 (FABP5) siRNA kit was purchased from RIBOBIO (Guangzhou). ShRNA plasmids or siRNA were transfected into breast cancer cells using Lipofectamine 2000 reagent (Invitrogen) according to the manufacturer’s instructions. ATGL stably silenced SUM159PT cells were selected with 2 μg/ml of puromycin (Sigma–Aldrich) for two weeks. siRNA transfected cells were used in the subsequent experiments at 48 h after transfection.

### Co-culture

Co-culture was performed using a Transwell system. Breast cancer cells were seeded (3.5 × 10^4^ SUM159PT cells or 10^5^ SK-BR-3 cells) in the inserts (0.4 μm polyester membrane; Corning Life Sciences, Lowell, MA) and cultivated alone or with mature adipocytes in the bottom chamber. In some cases, adipocytes were substituted for 0.02% Intralipid (Sigma–Aldrich) for 3 days.

### Bodipy staining and indirect immunofluorescence analysis

Intracellular lipids were examined by staining with Bodipy 493/503 (Invitrogen). Before co-culture, a glass coverslip was placed in each chamber. After the indicated co-culture time, cells on coverslips were fixed with 4% paraformaldehyde for 30 min at room temperature, and then permeabilized with 0.5% Triton X-100 for 5 min followed by incubation with Bodipy (15 min) and DAPI (5 min). After washing, the coverslips were mounted on slides with antifade mounting medium (Biosharp, Hefei, Anhui). Fluorescent images were acquired with a confocal laser microscopy system (Nikon A1plus Confocal Ti Microscope).

For the indirect immunofluorescence experiments, the cells were incubated with a primary antibody to E-cadherin (Santa Cruz, Shanghai Co., Ltd) overnight at 4 °C and then with the Alexa555 anti-mouse IgG secondary antibody (Invitrogen). The cells were then counterstained with DAPI and imaged with a confocal laser microscopy system.

### Triglyceride (TG) content measurement

Co-cultivated or non-co-cultivated breast cancer cells were harvested and lysed in 15 μl TG buffer (1 mM EDTA, 10 mM Tris-HCl, pH 7.5) [16]. TG content was quantified using an enzymatic colorimetric method (GPO-PAP reagent, Sichuan Marker Biotechnology, Sichuan) and read at a wavelength of 600 nm as described in the manufacturer’s protocol.

### Lipolysis and quantitative analysis of glycerol

SK-BR-3 or SUM159PT cells were grown with or without adipocytes for 3 days. The inserts were moved into new six-well plates and the medium was changed to serum-free DMEM to induce lipolysis. Then, cells were harvested at the indicated times to quantify intracellular TG content (0, 4, and 24 h) and the concentration of free glycerol (4 and 24 h) in the medium. A Free Glycerol Reagent kit (Sigma–Aldrich) was used to detect the released free glycerol.

### Cell proliferation and migration/invasion assays

Cellular proliferation was measured using a CellTiter 96 AQueous One Solution Cell Proliferation Assay kit (Promega, Madison, WI). A total of 2 × 10^3^ non-co-cultivated/co-cultivated cancer cells or transfected cancer cells were seeded onto 96-well plates in triplicate and the optical density was measured at 24, 48, and 72 h according to a protocol provided by the manufacturer.

Co-cultivated or non-co-cultivated breast cancer cells were fed with serum-free DMEM overnight before being trypsinized for migration and invasion assays. Tumor cells (5 × 10^4^) were allowed to migrate for 6 h (SUM159PT) or 24 h (SK-BR-3) in Transwell chambers (8 μm pore size, BD Biosciences, Franklin Lakes, NJ) towards a medium containing 10% (SUM159PT) or 20% (SK-BR-3) fetal bovine serum. For invasion assays, the Transwell chambers were pre-coated with Matrigel (0.1%, BD Bioscience) and cells were allowed to migrate for 24 h. After the indicated migration time, the Transwell membranes were fixed with 4% paraformaldehyde, followed by staining with 0.1% crystal violet. Cells on the inner surface were removed with cotton swabs. Invading cells on the outer surface were counted and photographed under an optical microscope in five fields per well.

#### RNA extraction and quantitative PCR

Total RNA was extracted using TRIzol reagent (Invitrogen) according to manufacture’s recommendation. The RNA concentration was measured by NanoDrop 2000 spectrophotometer (Thermo Scientific, Waltham, MA) and their quality was determined by gel electrophoresis. Reverse transcription was performed using Go-Taq polymerase (Promega, Madison, WI). A SYBR Green PCR Master Mix kit (Invitrogen) was used for quantitative RT-PCR in the 7500 Real-Time PCR System (Applied Biosystems, Foster City, CA). Relative expression levels of targeted genes were standardized to β-actin levels. Forward and reverse primers used are listed in Additional file 1: Table S1.

### Western blot analysis

Cells were washed twice and the total lysates were extracted with RIPA lysis buffer (Beyotime Institute of Biotechnology, Shanghai) containing protease inhibitor cocktail (Pierce, Cramlington, UK) with mixing by sonication. A bicinchoninic acid (BCA) standard curve was used to measure the lysate concentration. A total of 60 μg protein per sample was electrophoresed on SDS-polyacrylamide gels and transferred onto polyvinylidene fluoride membranes. After blocking with 5% skimmed milk in Tris-buffered saline, the membranes were incubated overnight at 4 °C with primary antibodies against: ATGL, phospho-AMP activated protein kinase (pAMPK), FABP5, ZO-1, Snail, MMP-2 and Erk1/2 bought from Cell Signaling Technology (Danvers, MA), hormone-sensitive lipase (HSL), AMPK and β-actin bought from Abcam (Cambridge, MA), and PPARβ/δ bought from Santa Cruze (Shanghai Co., Ltd). The following day, membranes were incubated with horseradish peroxidase-conjugated secondary antibodies. Antibody binding was visualized with an enhanced chemiluminescence detection system.

#### Immunohistochemistry

Normal breast tissues and primary breast tumor tissues from female patients were obtained from the First Affiliated Hospital of Chongqing Medical University. Informed consent was obtained from patients before sampling. Tissues were fixed in 10% neutral buffered formalin and embedded in paraffin. Sections (0.5 μm thick) were mounted on glass slides and deparaffinized in xylene, dehydrated in ethanol and rinsed in water. Antigen retrieval was performed with 0.01 M citrate buffer (pH 6.0) for 25 min in a steamer. The remaining steps were performed using a commercial kit (SP-9000, ZSGB-BIO, Beijing). Primary antibodies against ATGL (#2138, 1:800) and FABP5 (#39926, 1:100) were used, and PBS was used as an internal negative control. Staining was visualized by incubation in 3, 3-diaminobenzidine and counterstaining with hematoxylin. Slides were dehydrated and mounted with Histomount (ZLI-9550, ZSGB-BIO). Six randomly selected bright field microscopic images were captured using a LEICA DM500 microscope (Buffalo Grove, IL). Immunohistochemical staining intensity was scored as described by Pan et al. [19].

### Statistical analysis

Statistical analyses were performed with Graphpad Prism 6.0c (Graphpad Software, La Jolla, CA). The statistical significance of differences between means was evaluated using two-tailed Student’s *t*-tests; results are shown as the means ± SD. Representative results from at least three independent experiments are shown. The correlation between FABP5 expression and patient clinicopathological characteristics was analyzed by Chi-squared test. *P* values < 0.05 were deemed significant.

## Results

### Lipid accumulation and enhanced aggressiveness of breast cancer cells after co-culture

**Fig. 1:**
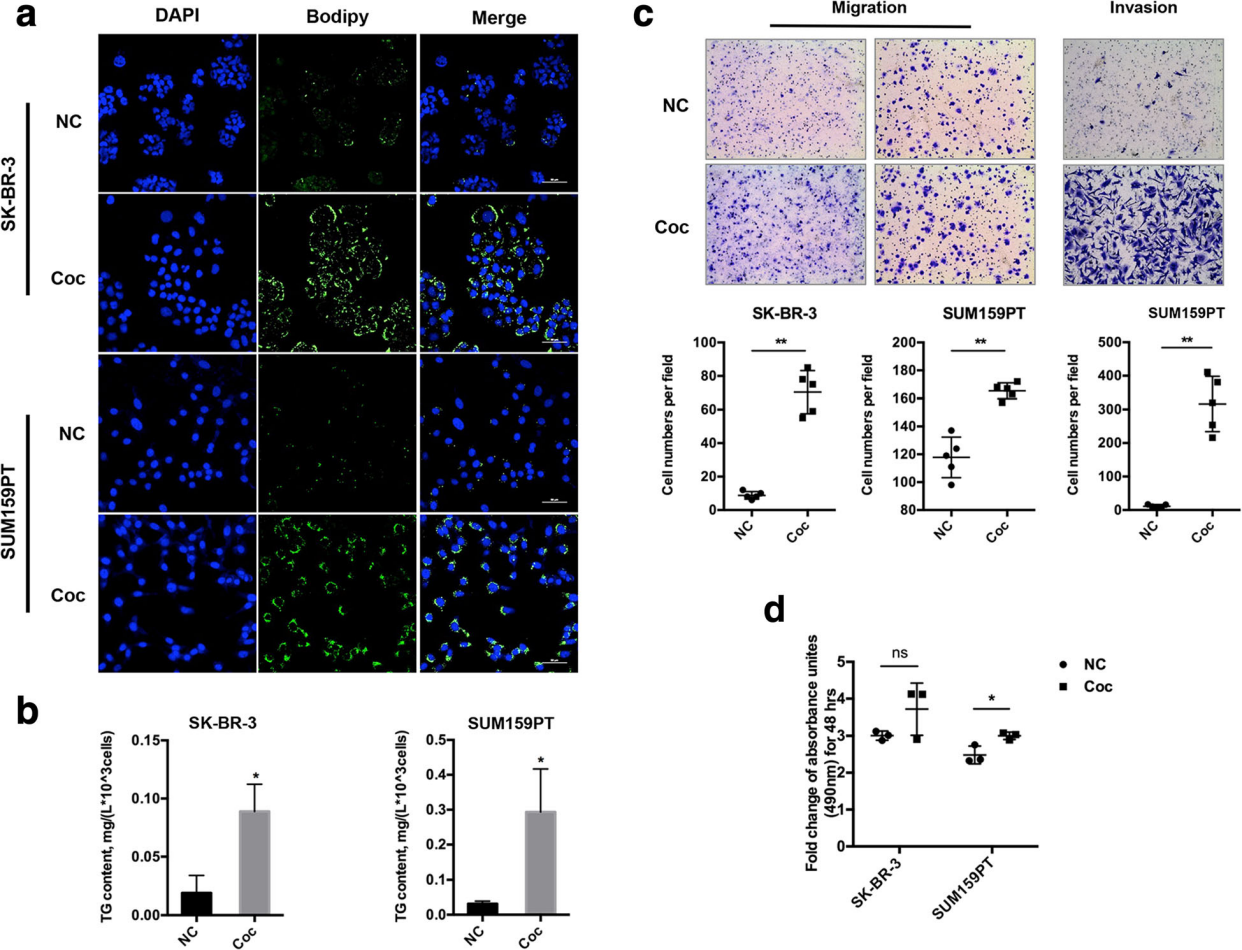
Lipid transfer during co-culture and co-cultivated breast cancer cells increased aggressiveness. **a** Lipid accumulation in cancer cells shown by Bodipy staining (lipids in green and nuclei in blue; scale bar, 50 μm), NC, non-co-culture; Coc, co-culture. **b** TG content in SK-BR-3 (left) and SUM159PT (right) cells cultured alone (NC) or with mature adipocytes (Coc) for 3 days. **c** Non-co-cultivated (NC) and co-cultivated (Coc) SK-BR-3 (left) and SUM159PT (right) cells migrated/invaded to the outer surface of the Transwell chamber; the migration times were 24 h and 6 h, respectively. Five fields were randomly taken for each chamber, and the representative migration images are shown. **d** Comparison of the proliferative ability of non-co-cultivated (NC) and co-cultivated (Coc) SUM159PT cells. Fold change comparison of the cell optical density during 48 h of SUM159PT cell non-co-cultivation (NC) or co-cultivation (Coc) with mature adipocytes for 3 days (*n* = 3). Representative results from at least three independent experiments are shown. *p < 0.05, **p < 0.01

The studies by Muller et al. and Balaban et al. observed a crosstalk between adipocytes and breast cancer cells during co-culture of the two cell populations. Lipid in adipocytes was mobilized, and the released free FAs were transferred into breast cancer cells to provide a metabolic substrate for tumor progression [8, 9, 15, 16]. We first reevaluated this phenomenon in our study. It has also been shown that excess intracellular FAs were esterified into TGs, a neutral lipid made up of three FAs esterified to the carbon backbone of a glycerol molecule, to protect against lipotoxicity [20]. Therefore, a fluorescent probe was employed to detect the accumulated neutral lipids in breast cancer cells. The results showed an intense increase in fluorescence intensity in co-cultivated SK-BR-3 and SUM159PT cells (Fig. 1a), which was paralleled by an apparent elevation in TG content in cancer cells (Fig. 1b). However, opposing changes were observed in adipocytes. After co-culture with breast cancer cells, lipid droplets in adipocytes became smaller both in size and quantity (Additional file 2: Figure S1).

To explore whether co-culture altered the biological behavior of breast cancer cells, comparative analyses of the malignant properties that represent growth and metastasis of cancer cells were conducted in non-co-cultivated and co-cultivated breast cancer cell populations. As shown in Fig. 1c, the migration capabilities of co-cultivated SK-BR-3 and SUM159PT cells were sharply increased. The invasion ability of SUM159PT cells was also tested, and the result was similar to the migration assay. However, co-culture had little influence on proliferation of the two recruited cancer cell lines (Fig. 1d). For SUM159PT cells, although the *p* value (0.0257) was statistically significant, the fold change of cell counts after 48 h showed little difference between co-cultivated and non-co-cultivated cells. In addition, the time frame was 6 h for the migration assay and 24 h for the invasion assay, so the enhanced migration and invasion properties of both cell lines were not attributed to increased cell number. Briefly, after co-culture with mature adipocytes differentiated from 3 T3-L1 cells, breast cancer cells were able to store more lipid and became more aggressive.

### Induced lipolysis in co-cultivated breast cancer cells is dependent on ATGL

**Fig. 2:**
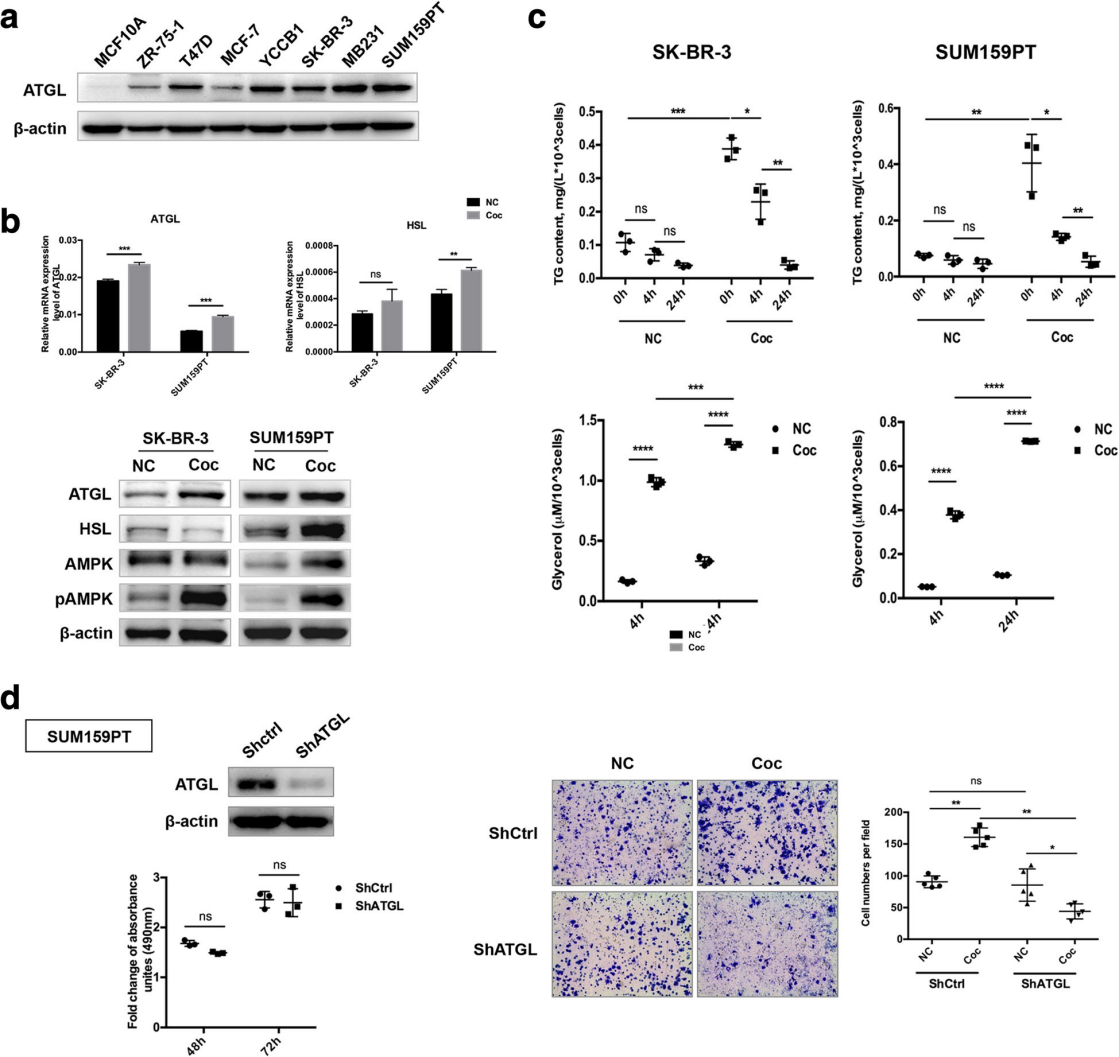
Co-culture with adipocytes induces ATGL-dependent lipolysis in breast cancer cells. **a** Expression of ATGL in mammary epithelial cell lines using western blotting. **b** After co-culture with mature adipocytes for 3 days, expression of ATGL in SK-BR-3 and SUM159PT cells was obviously increased. **c** Removing adipocytes and depriving serum from the medium led to lipolysis in cancer cells, and the TG content in tumor cells was detected at 0, 4 and 24 h after release, while free glycerol in the medium was detected at 4 and 24 h. **d** Down-regulation of ATGL in SUM159PT cells by shRAN plasmid transfection and validation of the effects using western blotting. Proliferative ability was compared between shCtrl and shATGL stably expressed SUM159PT cells (left panel). Transwell assays conducted to elucidate the impact of ATGL blockade on the migration ability of cancer cells. *p < 0.05, **p < 0.01, ***p < 0.001, ****p < 0.0001, “ns” stands for not significant

Lipolysis is the sequential hydrolysis of TG by ATGL, HSL and monoacylglycerol lipase (MAGL) [21]. ATGL is the rate-limiting lipase in this process [22] and accounts for approximately 70% of the activity in TG hydrolysis [23]. Thus, the cellular expression of ATGL was assayed by western blot. The results indicated that ATGL was barely expressed in the immortalized normal mammary epithelial cell line MCF-10A, however, for tumor cells, the expression level of ATGL was higher in the hormone receptor-negative cell lines SK-BR-3, MDA-MB231 and SUM159PT (Fig. 2a). A human epidermal growth factor receptor type 2 (HER2)-overexpression breast cancer cell line SK-BR-3 and a triple negative breast cancer (TNBC) cell line SUM159PT were recruited for the subsequent experiments. After being grown in the presence of mature adipocytes, the expression of the two major lipases, ATGL and HSL, increased in SK-BR-3 and SUM159PT cells (Fig. 2b), indicating an elevated lipolytic effect. Simultaneously, the central metabolic sensor AMPK and its activated form pAMPK were also detected. Upregulation of the pAMPK/AMPK ratio suggested that dynamic catabolism in cancer cells was stimulated (Fig. 2b). In agreement with the results from the cellular experiments, the expression of ATGL was determined in breast tissue specimens showed a similar trend (Additional file 3: Figure S2). ATGL was not expressed in normal breast but was present in breast cancer. Notably, tumor cells in the proximity of adipose tissue displayed upregulated ATGL expression versus more distant areas, as shown by the red arrow points. Additionally, adipocytes in contact with tumor cells (red asterisk) showed a smaller cellular size compared to cells more distant from the tumor (black asterisk), suggesting a loss of cellular contents. Moreover, results from Wang et al. implied a close correlation between the expression of ATGL and HER2 [16].

We then questioned whether the stored TG in tumor cells was mobilized to provide substrates for enhanced aggressive activities. In addition to the expression levels of lipases, the quantity of TG in tumor cells and the released free glycerol in the medium were also detected at different time points after moving cancer cells from the co-culture system. Over time, the cancer cells were able to use the stored lipids, as revealed by the gradual loss of TG content in both SK-BR-3 and SUM159PT cells (Fig. 2c, upper panel). Conversely, released glycerol in the culture medium was increased (Fig. 2c, lower panel). Notably, the lipolytic rates of cancer cells cultivated with adipocytes were remarkably improved compared to cells grown alone.

To provide further evidence that the increased migration and invasion capabilities of breast cancer cells were dependent on the hydrolysis and utilization of adipocyte-derived lipids, cell functional assays were conducted in which ATGL was stably eliminated by shRNA vector transfection (Fig. 2d). Excluding differences in cell proliferation, silencing of ATGL dramatically impaired the migration ability of co-cultivated SUM159PT cells. Therefore, we conclude that the increased aggressiveness of breast cancer cells was ATGL-dependent after co-culture with adipocytes.

### Acceleration of fatty acid trafficking in cancer cells is mediated by FABP5

Whether the FAs liberated from adipocytes were being transferred into breast cancer cells for storage as TGs, or the FAs released from hydrolysis of the stored TGs are transported to specific organelles for further use, the mechanism of how FAs precisely and efficiently shuttle remains unknown. The fatty acid binding proteins (FABPs) family is a category of transport proteins that includes nine isoforms specific to various tissues and substrates with high affinity for long-chain FAs, bile acids and retinoids [24, 25]. FABP5 is a 15-kDa protein that belongs to the FABPs family. It is involved in the uptake and transport of long-chain FAs and plays a key role in cell signaling, gene regulation, cell growth and differentiation [26]. FABP4 and FABP5 are highly homologous and bind to FAs with similar selectivity and affinity. They have redundant and overlapping roles in mediating FA uptake in various tissues [27]. In addition, FABP4 has been reported to mediate the transfer of FAs from adipocytes into cancer cells during the cancer cell-adipocyte interaction [5, 13, 28].

**Fig. 3:**
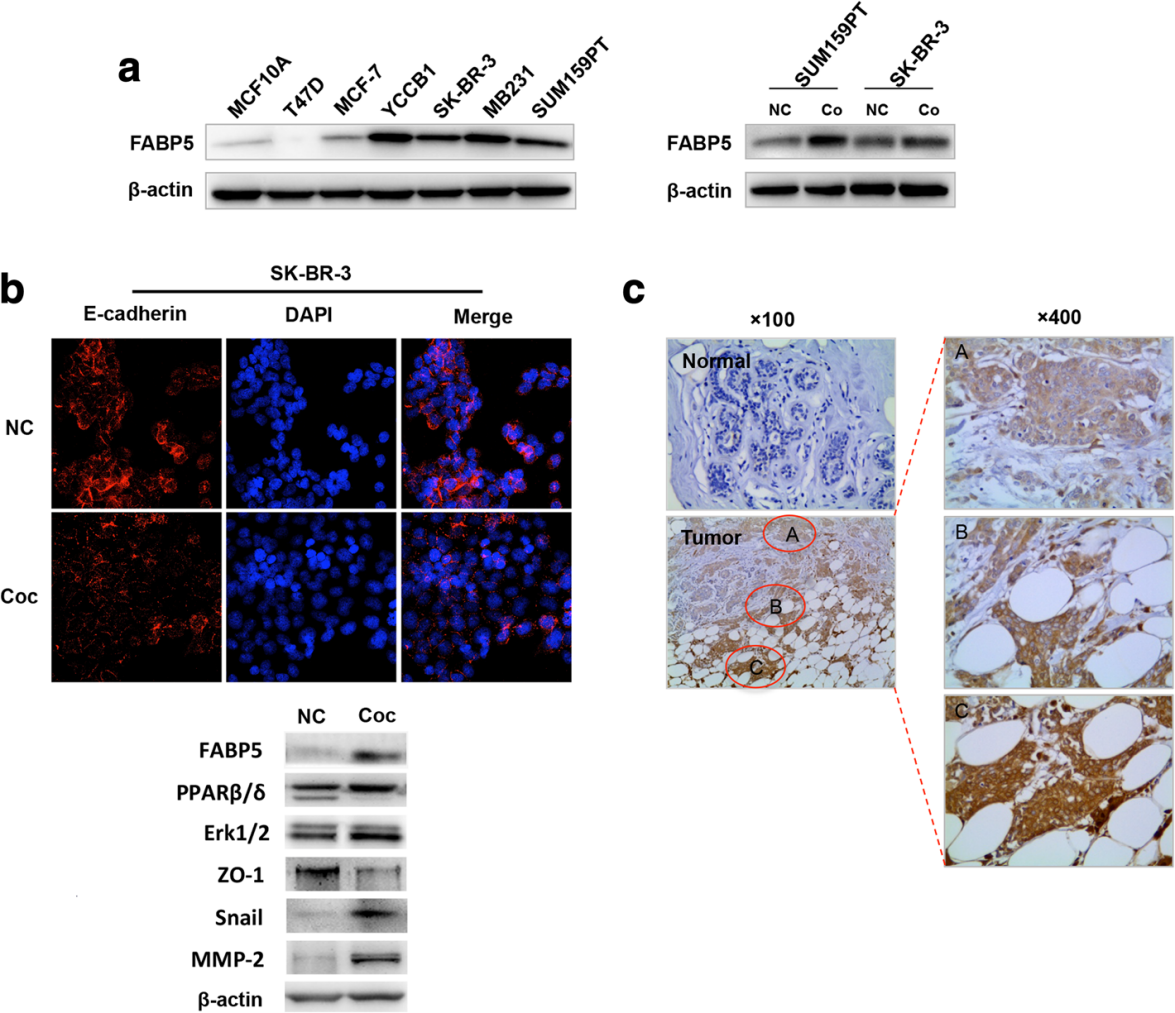
Elevated expression of FABP5 induce EMT inbreast cancer cells. **a** Expression of FABP5 in different mammary epithelial cell lines (left panel) and in non-co-cultivated (NC) and co-cultivated (Coc) breast cancer cells (SUM159PT and SK-BR-3, right panel). **b** Detection of E-cadherin in the SK-BR-3 breast cancer cells by immunofluorescence analysis (upper panel) and some molecules that may contribute to the enhance malignancy of breast cancers by western blot (lower panel). **c** Representative immunohistochemical staining of FABP5 in normal breast and tumor tissues

First, the mRNA expression levels of FABP4,FABP5 and another putative FA translocase, CD36 [29], were detected in various breast cancer cell lines. Our results revealed that only FABP5 was expressed at a considerable level in breast cancer cells (Additional file 4: Figure S3A). This was in accordance with Guaita-Esteruelas’s study in which FABP4 and CD36 were found to be rarely expressed in the breast cancer cell lines MCF-7 and MDA-MB231 when detected by western blot [30]. Consequently, FABP5 in different mammary epithelial cell lines was assessed; FABP5 was more highly expressed in breast cancer cell lines with more aggressive phenotypes (Fig. 3a, left panel). Importantly, co-culture also increased FABP5 expression in SK-BR-3 and SUM159PT cells (Fig. 3a, right panel). PPARβ/δ has been shown to be expressed in different types of cancer and mediate tumorigenesis, drug resistance and cancer metastasis through transcriptionally activating many kinds of growth factor signaling pathways. On the other hand, FABP5 has been reported to enhance the metastatic potential and tumorigenesis through activation of EGFR signaling pathway in breast cancer [31, 32] and induce the epithelial-mesenchymal transaction (EMT) in hepatocellular cancer [33]. Thus, we deduced a /PPARβ/δ/EGFR/MAPK pathway was activated by redundant fatty acids being transported into nucleus via upregulated expression of FABP5 after co-culture with mature adipocytes in this study. The HER2-overexpression breast cancer cell line SK-BR-3 was chosen to verify the hypothesis and some putative EMT markers in breast cancer cells were detected after co-culture. As shown in Fig. 3b, the decreased expression of epithelial markers (E-cadherin and ZO-1) and increased expression of mesenchymal marker (Snail) were observed, as well as the activation of PPARβ/δ and MAPK.

In parallel with the expression pattern in tumor cells, FABP5 was not expressed in normal breast tissues, but in cancerous specimens. The closer to adipose tissue the cancer, the higher expression of FABP5 (Fig. 3c). The above results imply a close relationship between the expression of FABP5 in breast cancer cells and the presence of adipocytes in the microenvironment. Additionally, FABP5 was stained in a total of 60 paraffin-embedded tumor sections, and the correlation between staining intensity and patient clinicopathological information was analyzed. The data showed that high expression of FABP5 tended to distribute in grade III, estrogen receptor negative and triple negative breast cancer patients (*p* = 0.036, 0.032 and 0.001, respectively). The detailed FABP5 staining intensity distribution in various breast cancer patients classified by clinicopathological features are listed in Table 1.

**Table 1:** Immunohistochemistry staining intensity of FABP5 in various breast cancer patients

Clinicopathological features	Expression (*N* = 60)		*p* value
	- ~Medium n (%)	High n (%)	
Age, years			0.166
≤ 50	25 (49.0)	2 (22.2)	
> 50	26 (51.0)	7 (77.8)	
BMI ^a^ (Kg/m^2^)			1.000
18.5–24.9	33 (78.6)	6 (75.0)	
≥ 25	9 (21.4)	2 (25.0)	
Tumor size			0.712
≤ 2 cm	18 (35.3)	4 (44.4)	
> 2 cm	33 (64.7)	5 (55.6)	
Grade			0.036
I-II	41 (80.4)	4 (44.4)	
III	10 (19.6)	5 (55.6)	
Lymph node metastasis			1.000
Negative	28 (54.9)	5 (55.6)	
Positive	23 (45.1)	4 (44.4)	
Estrogen receptor			0.032
Negative	19 (37.3)	7 (77.8)	
Positive	32 (62.7)	2 (22.2)	
Progesterone receptor			0.281
Negative	28 (54.9)	7 (77.8)	
Positive	23 (45.1)	2 (22.2)	
Ki67			0.704
Low	18 (35.3)	2 (22.2)	
High	33 (64.7)	7 (77.8)	
P53			1.000
Negative	19 (37.3)	3 (33.3)	
Positive	32 (62.7)	6 (66.7)	
HER2^b^			0.225
Negative	23 (57.5)	6 (85.7)	
Positive	17 (42.5)	1 (14.3)	
Subtypes^b^			0.001
TNBC	6 (15.0)	6 (85.7)	
Non-TNBC	34 (85.0)	1 (14.3)	

^a^Information on some patients was not well documented.^b^HER2 status was detected using Fluorescence in Situ Hybridization (FISH) and patients with unknown HER2 status were not included in the statistical analysis

**Fig. 4:**
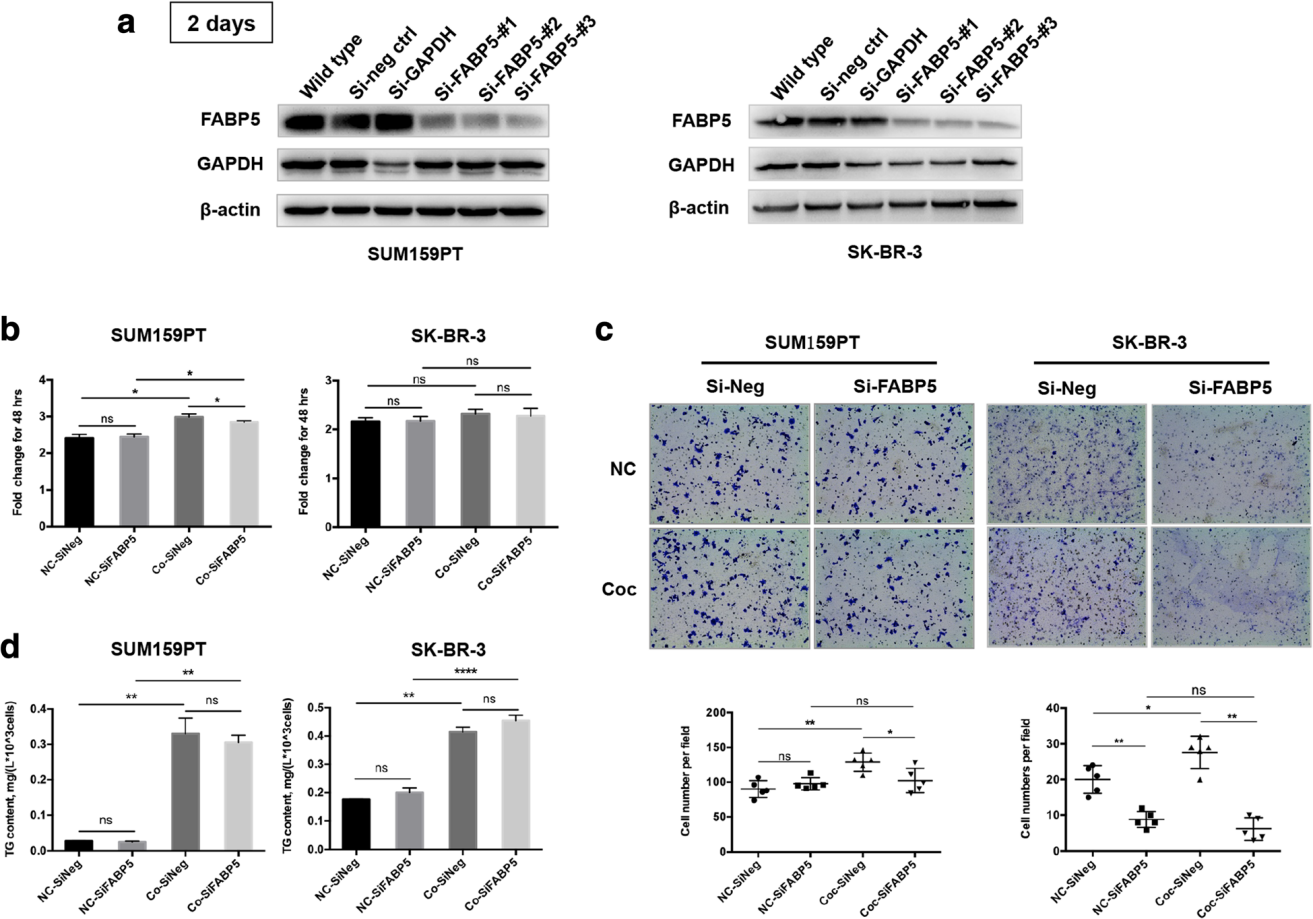
In the presence of adipocytes, accelerated FA trafficking in breast cancer cells was mediated by FABP5. **a** After ablation of FABP5 using an siRNA kit, cells were harvested 48 h after transfection for western blotting to the validate knockdown effectiveness. **b** Impact on the proliferative ability of breast cancer cell lines resulting from FABP5knockdown. **c** Altered migration ability resulting from FABP5 deletion in non-cultivated (NC) and co-cultivated (Coc) cancer cells. **d** TG storage in breast cancer cells, with FABP5 expressed or ablated, cultivated in the absence or presence of adipocytes. *p < 0.05, **p < 0.01, ****p < 0.0001, “ns” stands for not significant

To deeply elucidate the key role of FABP5 in the breast cancer cell-adipocyte interaction, FABP5 was successfully blocked by transfection of targeting siRNA (Fig. 4a and Additional file 4: Figure S3B). Blockade of FABP5 exerted no obvious impact on the proliferative ability of SUM159PT cells (after 48 h of growth the fold change in cell number was still less than 1, although the *p* value was significant) (Fig. 4b). However, deficiency of FABP5 seriously crippled the migration ability of tumor cells, especially after co-culture (Fig. 4c). FABP5 is a FA transporter involved in the uptake and transport of long chain FAs. Ablation of FABP5 probably affects TG storage in cancer cells since FA afflux may be hindered. Thus, TG content was detected and the results support the conclusion that FABP5 was more likely to participate in intracellular FA trafficking rather than transmembrane transportation, since down-regulation of FABP5 did not reduce lipid accumulation in cancer cells during co-culture (Fig. 4d). Generally, co-culture with adipocytes upregulated the expression of FABP5 in tumor cells to ensure efficient FA transportation to provide substrate for downstream applications, either oxidation and energy supply, or to participate in signaling, like inducing EMT, as discovered in this study.

### Adipocyte-breast cancer cell crosstalk, but not lipid alone, contributes to cancer cell progression

**Fig. 5:**
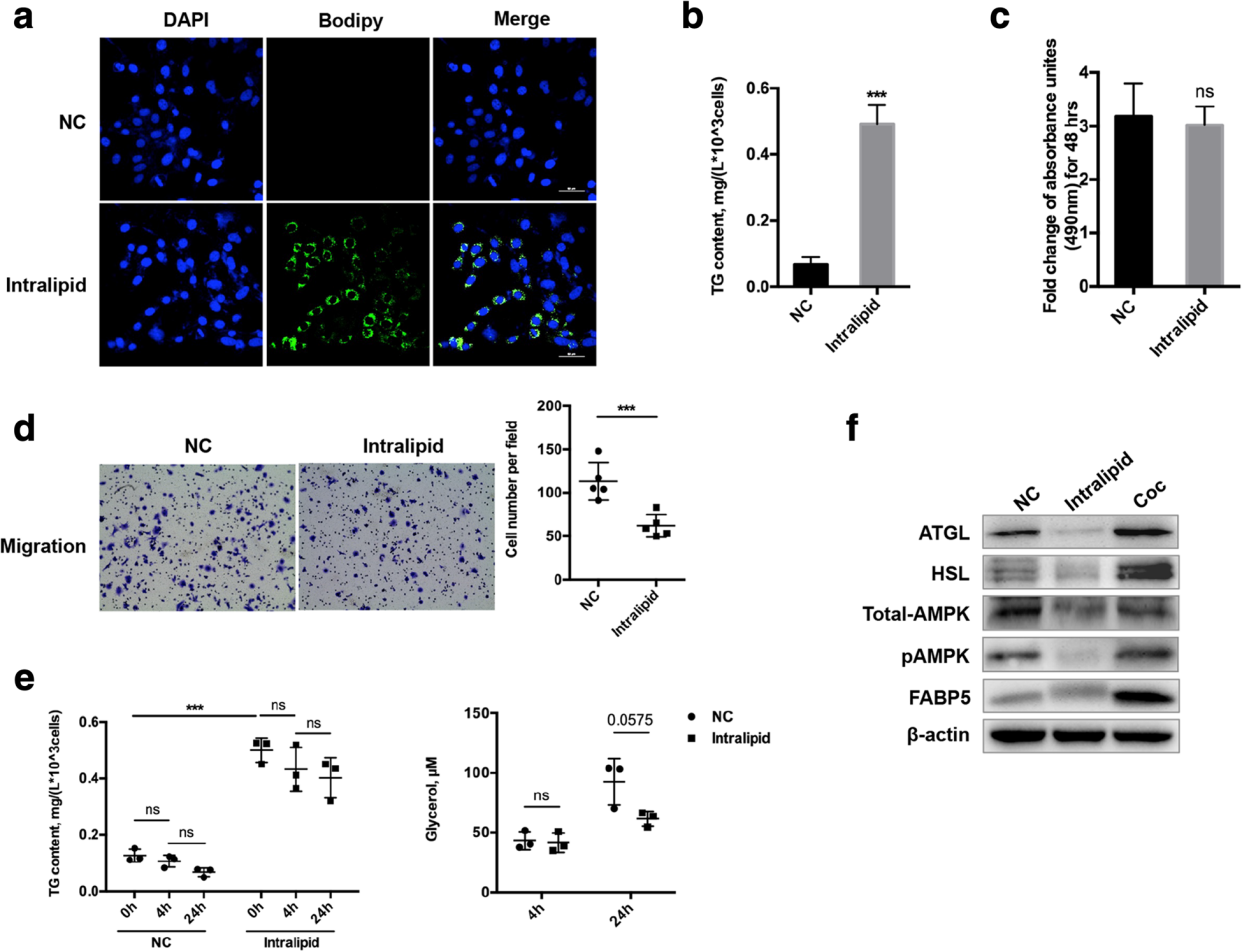
Exogenous lipids in the absence of adipocytes could not promote the malignant behavior of cancer cells. Mature adipocytes were replaced by 0.02% Intralipid in 3 day co-culture with SUM159PT breast cancer cells. **a** Bodipy staining was performed on SUM159PT cells grown alone (NC) and in medium supplemented with Intralipid. (lipids are shown in green, and nuclei are shown in blue; scale bar, 50 μm). **b** The TG content in the two cell populations (NC vs. Intralipid) was measured. **c-d** Proliferation and migration assays were employed to compare the malignant properties of non-treated cells (NC) and Intralipid-treated cells. **e** SUM159PT cells were not treated (NC) or treated with Intralipid for 3 days and then released similar to co-culture. Cells were collected at 0, 4 and 24 h for TG measurement, and medium was collected at 4 and 24 h for free glycerol measurement. **f** Differential expression of lipases, pAMPK/AMPK and FABP5 in breast cancer cells (SUM159PT) grown in Intralipid or with adipocytes. ***p < 0.001, “ns” means not significant

To evaluate the vital function of adipocytes in the induction of lipid metabolism in breast cancer cells, an exogenous lipid emulsion was added into the cancer cell medium to replace mature adipocytes. As shown in Fig. 5, after cultivation in medium containing 0.02% Intralipid, lipids were markedly increased in SUM159PT cells, in parallel to increased intracellular TG content (Fig. 5a, b). Nevertheless, increased TG content did not correlate with improved proliferation and migration capacities of cancer cells (Fig. 5c, d). This may be attributable to dormant lipolysis and inactive utilization of FAs in cancer cells, since with increased release time, only a very small amount of the stored TG was hydrolyzed into FAs and free glycerol (Fig. 5e). Further, the expression of ATGL and HSL were negligibly elevated, as was the intracellular FA trafficking protein FABP5 (Fig. 5f). Collectively, these results indicate that reciprocal stimulation of adipocytes and breast cancer cells plays an important role in promoting cancer cell progression.

## Discussion

The tumor microenvironment has been increasingly recognized as a major regulator of carcinogenesis. A growing number of studies have focused on the tumor environment to explore the complex mechanisms underlying tumorigenesis and disease progression. The breast is a fat-rich organ. Invasive breast tumors grow in a microenvironment surrounded by adipocytes. Awareness of the crosstalk between the breast tumor and adipose tissue is providing a new and crucial therapeutic target. Our current results, in accordance with reports by Balaban et al. and Wang et al. [15, 16], demonstrate that breast cancer cells can induce lipolysis in adipocytes and that the FAs released by adipocytes may translocate into tumor cells for further use. This bidirectional metabolic interaction was associated with increased migration and invasion capabilities of breast cancer cells. Moreover, the biological behavioral changes in breast cancer cells did not occur by feeding cancer cells an exogenous fat emulsion instead of co-culture with adipocytes. It is noteworthy that in this study, we discovered the protumoral role of FABP5 through intracellular FA transportation and signaling activation in breast cancer cells with the presence of adipocytes.

FAs contribute to cancer progression by multiple mechanisms. For example, they act as building blocks for membrane phospholipids, supply energy via β-oxidation and are used for the biosynthesis of pro-tumorigenic lipid-signaling molecules. In most normal cells, de novo FA synthesis is suppressed, but is upregulated in tumor cells. Additionally, cancer cells can incorporate and utilize exogenous lipids to substitute for endogenously derived FAs to promote cell viability [34]. In fact, lipogenesis and lipolysis coexist in cancer cells, and a balance between lipogenesis, lipid uptake and lipolysis is required to maintain lipid homeostasis [35]. During co-culture, induced lipolysis in adipocytes releases abundant FAs, which may serve as a lipid reservoir for breast cancer cells. After being transferred into breast cancer cells, a portion of FAs then participate in elevated β-oxidation. The excess FAs are transformed into TGs and stored as lipids to protect against lipotoxicity, which could lead to apoptosis of non-adipose cells [16, 20]. In accordance with these findings, lipid accumulation and increased TG content in co-cultivated breast cancer cells were observed in this study.

Lipid mobilization or lipolysis involves one molecule of TG being sequentially hydrolyzed by ATGL, HSL and MAGL to liberate three molecules of free FAs and one molecule of glycerol [21]. As previously mentioned, ATGL is the essential lipase in TG hydrolysis [22]; ATGL and HSL are responsible for 95% of the hydrolytic activity in lipolysis [23]. After co-culture of SUM159PT or SK-BR-3 cells with adipocytes, increased expression of ATGL and HSL was detected, as well as diminished TG content and increased release of free glycerol over time, implying induction of lipolysis and ATGL-mediated utilization of accumulated lipids, which were mainly used to promote tumor invasion. Invasion is an energy-consuming process and breast cancer cells urgently demand ATP. The elevated ratio of AMP/ATP or ADP/ATP could activate AMPK, the key sensor of cellular energy status [36], in co-cultivated breast cancer cells in this current study.

Hydrolysis of stored TGs produces ample FAs. Not only are FAs important energy sources, they are also signaling molecules that may stimulate nuclear peroxisome proliferative-activated receptors (PPARs), which are ligand specific transcription factors [37]. FABPs are involved in binding and sorting free FAs, as well as transporting them to their appropriate compartments within the cell. Among then, FABP4 and FABP5 are the most recognized members. As described above, FABP4 and CD36 were scarcely expressed in breast cancer cell lines in the current study, so we focused on FABP5. One study after another has reported that FABP5 could transport large amounts of intracellular FAs into nucleus to activate PPARs, which may then activate downstream cancer-promoting genes [38–40]. In the present study, FABP5 was scarcely expressed in the normal breast epithelial cell line MCF10A but was highly expressed in cancer cell lines. High levels of expression were found in hormone receptor-negative cell lines. Moreover, the presence of adipocytes obviously increased the expression of FABP5. Data from cellular experiments were verified in tissue assays. FABP5 was not expressed in normal breast tissues. In tumor sections, the highest expression was observed at the border between tumor cells and adipocytes. Furthermore, a higher expression level of FABP5 was found in high grade tumors and TNBC, which are serious factors for poor prognosis. Our data are in agreement with those of Liu et al. They discovered that the expression level of FABP5 was higher in estrogen and progesterone negative breast cancer, with the highest expression in TNBC. Elevated expression of FABP5 correlated with high tumor grade and poor prognosis [41]. Our migration assay in vitro revealed an exaggerated difference between FABP5-expressed and FABP5-down-regulated cells after co-culture. Knockdown of FABP5 significantly weakened the migration ability of co-cultivated cancer cells, implicating a critical impact from the surrounding adipocytes. Further experiments of immunofluorescence assay and western blotting partially revealed that, the interaction with adipocytes promoted transportation of FAs into nucleus in breast cancer cells by upregulated FABP5 to activate PPARβ/δ, then induce EMT and secretion of MMP-2 to facilitate migration ad invasion possibly through activation of EGFR/MAPK signaling pathway.

While our current study supplements previous findings, how adipocytes promote the malignant progression of breast cancer cells via upregulated FABP5 has not been thoroughly elucidated, but subsequent research is now in progress. In our future work, some reported mechanisms in which FABP5 participates could be used for reference. For instance, FABP5 might be involved in MAGL-dependent signaling to regulate a FA network that promotes migration, invasion, and survival of cancer cells [42, 43]. Reportedly, MAGL is highly expressed in aggressive human cancer cells and primary tumors, and promotes migration, invasion, survival, and in vivo tumor growth by regulating a FA network enriched in oncogenic signaling lipids [43]. Kawaguchi et al. discovered that knockdown of FABP5 significantly suppressed HSL and MAGL, likely leading to the decreased invasion potential of colorectal cancer cells HCT116 [42]. In our study, both the rate-limiting lipase ATGL and FA transporter FABP5 acted as accelerators of breast cancer, and the latent relationship between these two molecules deserves further investigation.

## Conclusions

Adipocyte-breast cancer cell crosstalk facilitates metabolic modification of breast cancer cells. Breast cancer cells become capable of utilizing FAs that originate from surrounding adipocytes. Elevated expression of ATGL and FABP5 play critical roles during this process. Targeting ATGL and FABP5 could provide new therapeutic strategies for patients with breast cancer.

## Additional files

Additional file 1: **Table S1.** List of primers used in this study. (PDF 63 kb)

Additional file 2: **Figure S1.** Lipid in adipocytes cultured in the absence (NC) or presence (Coc) of SUM159PT cells for 3 days (upper panel, Bodipy staining; lower panel, oil red O staining). (PDF 911 kb)

Additional file 3: **Figure S2.** Representative immunohistochemical staining of ATGL in normal breast and tumor tissues. (PDF 2736 kb)

Additional file 4: **Figure S3.** (A). Relative mRNA expression levels of FABP4, FABP5 and CD36 in breast cancer cell lines. (B). Tumor cells were transfected with FABP5-targeting siRNA, and cells were harvested at day 6 after transfection for western blotting. (PDF 362 kb)

## Abbreviations

ADP: Adenosine diphosphate
AMP: Adenosine monophosphate
AMPK: AMP activated protein kinase
ATGL: Adipose triglyceride lipase
ATP: Adenosine triphosphate
EGFR: Epidermal growth factor receptor
EMT: Epithelial-mesenchymal transaction
FABP4: Fatty acid binding protein 4
FABP5: Fatty acid binding protein 5
FAO: Fatty acid oxidation
FAs: fatty Acids
HER2: Human epidermal growth factor receptor type 2
HSL: Hormone-sensitive lipase
MAGL: Monoacylglycerol lipase
MAPK: Mitogen-activated protein kinase
MMP-2: Matrix metalloproteinase 2
PPARs: Peroxisome proliferative-activated receptors
TG: Triglyceride.
TNBC: Triple negative breast cancer
ZO-1: Zonula occludens-1
