# Supplementary material for: Utilization of adipocyte-derived lipids and enhanced intracellular trafficking of fatty acids contribute to breast cancer progression
Source: Cell Commun Signal. 2018 Jun 18;16:32. doi: 10.1186/s12964-018-0221-6 (PMC6006729; doi:10.1186/s12964-018-0221-6)
Supplement: Supplementary file 1 — Table S1. List of primers used in this study. (PDF 63 kb) [file 12964_2018_221_MOESM1_ESM.pdf]

Additional file 1

Table S1: List of primers used in this study.

Primers	Sequence (5'-3')	Primers	Sequence (5'-3')
ATGL-F	AAAGATCATCCGCAGTTTCC	*ATGL*-R	CCTTGGAGTTGAAGTGGGAT
HSL-F	TGCGTGAAGGACAGGACAG	*HSL*-R	AGGTAGGGCTCGTGGGAT
CD36-F	TGAACAGCAGCAACATTCAA	*CD36*-R	GCTGCAGGAAAGAGACTGTG
FABP5-F	TACCCTGGGAGAGAAGTTTGA	*FABP5*-R	ATTGTGCTTTCCTTCCCATC
FABP4-F	GGTACATGTGCAGAAATGGG	*FABP4*-R	TCCCTTGGCTTATGCTCTCT
β-actin-F	TCCTGTGGCATCCACGAAACT	β-actin-R	GAAGCATTTGCGGTGGACGAT
