# Supplementary material for: Utilization of adipocyte-derived lipids and enhanced intracellular trafficking of fatty acids contribute to breast cancer progression
Source: Cell Commun Signal. 2018 Jun 18;16:32. doi: 10.1186/s12964-018-0221-6 (PMC6006729; doi:10.1186/s12964-018-0221-6)
Supplement: Supplementary file 2 — Figure S1. Lipid in adipocytes cultured in the absence (NC) or presence (Coc) of SUM159PT cells for 3 days (upper panel, Bodipy staining; lower panel, oil red O staining). (PDF 911 kb) [file 12964_2018_221_MOESM2_ESM.pdf]

**Additional file 2**

**Oil Red O staining**

Non-cocultivated and cocultivated adipocytes were fixed in 4% paraformaldehyde for 30 min at room temperature, rinsed twice with phosphate-buffered saline and air-dried. The cells were then stained for 20 min with Oil Red O (Sigma–Aldrich, St. Louis, MO) in 60% (v/v) isopropanol and washed. Images were photographed immediately using an inversion fluorescence microscope (LEICA DMI4000).

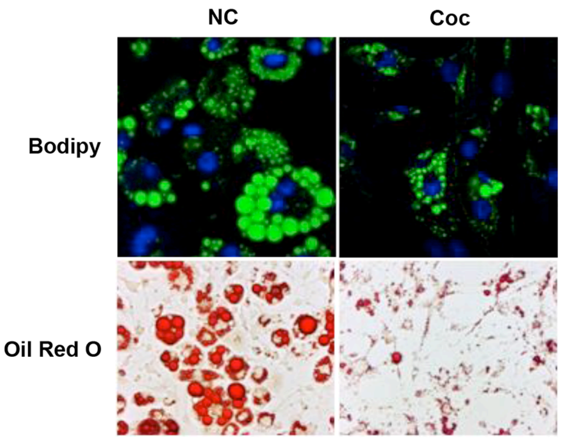


**Fig S1.** Lipid in adipocytes cultured in the absence (NC) or presence (Coc) of SUM159PT cells for 3 days (upper panel, Bodipy stainning; lower panel, oil red O staining).
