# Supplementary material for: Utilization of adipocyte-derived lipids and enhanced intracellular trafficking of fatty acids contribute to breast cancer progression
Source: Cell Commun Signal. 2018 Jun 18;16:32. doi: 10.1186/s12964-018-0221-6 (PMC6006729; doi:10.1186/s12964-018-0221-6)
Supplement: Supplementary file 3 — Figure S2. Representative immunohistochemical staining of ATGL in normal breast and tumor tissues. (PDF 2736 kb) [file 12964_2018_221_MOESM3_ESM.pdf]

Additional file 3

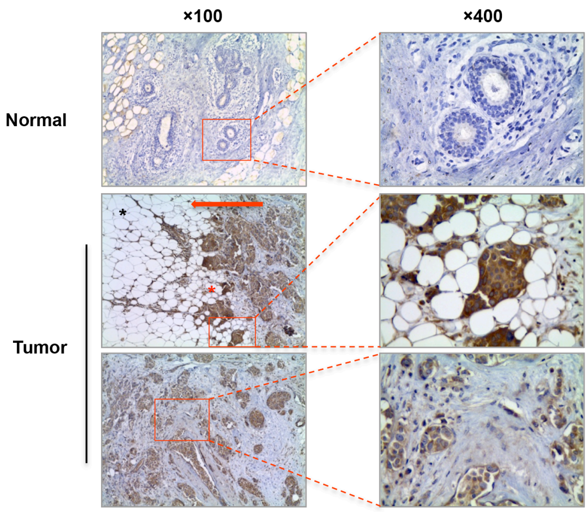

Fig S2. Representative immunohistochemical staining of ATGL in normal breast and tumor tissues.
