# Supplementary material for: Utilization of adipocyte-derived lipids and enhanced intracellular trafficking of fatty acids contribute to breast cancer progression
Source: Cell Commun Signal. 2018 Jun 18;16:32. doi: 10.1186/s12964-018-0221-6 (PMC6006729; doi:10.1186/s12964-018-0221-6)
Supplement: Supplementary file 4 — Figure S3. (A). Relative mRNA expression levels of FABP4, FABP5 and CD36 in breast cancer cell lines. (B). Tumor cells were transfected with FABP5-targeting siRNA, and cells were harvested at day 6 after transfection for western blotting. (PDF 362 kb) [file 12964_2018_221_MOESM4_ESM.pdf]

Additional file 4

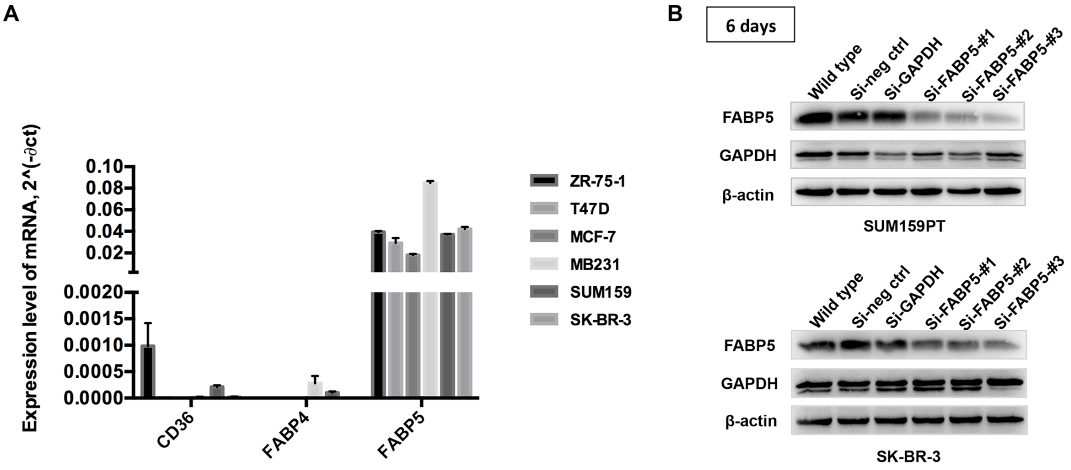

Fig S3. (A). Relative mRNA expression levels of FABP4, FABP5 and CD36 in breast cancer cell lines. (B). Tumor cells were transfected with FABP5-targeting siRNA, and cells were harvested at day 6 after transfection for western blotting assay.
